# Nanocomposite Based on HA/PVTMS/Cl_2_FeH_8_O_4_ as a Gas and Temperature Sensor

**DOI:** 10.3390/s222410012

**Published:** 2022-12-19

**Authors:** Sohrab Nasiri, Marzieh Rabiei, Ieva Markuniene, Mozhgan Hosseinnezhad, Reza Ebrahimi-Kahrizsangi, Arvydas Palevicius, Andrius Vilkauskas, Giedrius Janusas

**Affiliations:** 1 Faculty of Mechanical Engineering and Design, Kaunas University of Technology, Studentu Street 56, 51373 Kaunas, Lithuania; 2 Department of Organic Colorants, Institute for Color Science and Technology, Tehran P.O. Box 16656118481, Iran; 3 Advanced Materials Research Center, Department of Materials Engineering, Najafabad Branch, Islamic Azad University of Najafabad, Najafabad P.O. Box 8514143131, Iran

**Keywords:** nanocomposite, HA/PVTMS/C_l2_FeH_8_O_4_, sol–gel, freeze-dryer, sensor

## Abstract

In this paper, a novel nanocrystalline composite material of hydroxyapatite (HA)/polyvinyltrimethoxysilane (PVTMS)/iron(II)chloride tetrahydrate (Cl_2_FeH_8_-O_4_) with hexagonal structure is proposed for the fabrication of a gas/temperature sensor. Taking into account the sensitivity of HA to high temperatures, to prevent the collapse and breakdown of bonds and the leakage of volatiles without damaging the composite structure, a freeze-drying machine is designed and fabricated. X-ray diffraction, FTIR, SEM, EDAX, TEM, absorption and photoluminescence analyses of composite are studied. XRD is used to confirm the material structure and the crystallite size of the composite is calculated by the Monshi–Scherrer method, and a value of 81.60 ± 0.06 nm is obtained. The influence of the oxygen environment on the absorption and photoluminescence measurements of the composite and the influence of vaporized ethanol, N_2_ and CO on the SiO_2_/composite/Ag sensor device are investigated. The sensor with a 30 nm-thick layer of composite shows the highest response to vaporized ethanol, N_2_ and ambient CO. Overall, the composite and sensor exhibit a good selectivity to oxygen, vaporized ethanol, N_2_ and CO environments.

## 1. Introduction

**Figure 1:**
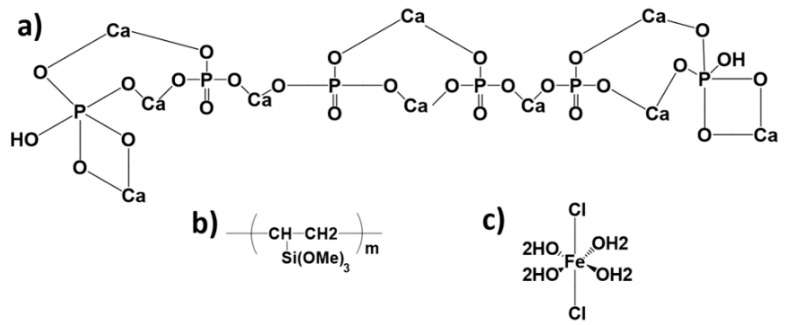
The chemical structures of the composite components (**a**) HA, (**b**) PVTMS and (**c**) Cl_2_FeH_8_O_4_.

Researchers have constantly strived to improve the quality of sensors in terms of sensitivity, durability and accuracy for their chosen applications. With the rapid improvement in the economy, the number of sensor composites developed by industry has also increased. Sensors are generally the interface of an electrical control system to the environment. The sensor converts information from the environment such as pressure, temperature, force, light, etc., into an electrical signal [1]. A sensor is always a part of a data acquisition system. Often, such a system is part of a larger control system that includes various framework mechanisms [2]. Semiconductor metal oxide gas sensors usually operate at high temperatures and have low sensitivity [3]. For this reason, intensive advanced techniques have been reported to improve gas sensor performance, such as aliovalent doping [4], UV light illumination [5] and noble metal loading [6]. Increasing concern for the protection of the environment has led to the continued development of gas sensors. The importance of these sensors is well known, and much research is being done to develop suitable, gas-sensitive materials [7]. Among the existing gas sensors, resistive gas sensors have become very important for the detection of combustible and toxic gases in recent decades [8,9,10]. In particular, conductive polymers are widely used as gas sensors due to their unique properties, such as high sensitivity, low cost and easy fabrication [11]. Calcium phosphates come in various forms that have different crystal structures and Ca/P ratios, such as hydroxyapatite (HA), octacalcium phosphate, tricalcium phosphate, dicalcium phosphate dihydrate and dicalcium phosphate [12]. One of the most well-known calcium phosphate groups is HA. HA is a versatile biomaterial with the chemical composition Ca_10_(PO_4_)_6_(OH)_2_, and has important applications in biomedical engineering, such as in bone scaffolds, drug delivery systems, dental implants, bone fillers, implant coatings and chromatography (protein processing) [13]. Biological HA differs from mineral HA; it consists of many deviations, such as non-stoichiometric parts, substitutions, voids and other defects [14]. It is also widely used as a sensor in doped and compound forms for analytes [15]. Several methods have been described so far for the synthesis of HA nanoparticles, including wet chemical sol–gel, hydrothermal, heat treatment and microwave methods [16,17,18]. Furthermore, doping HA with iron (Fe) has resulted in a strong ferromagnetic material that has found applications in magnetic resonance imaging, drug delivery, cell separation, and as a heat mediator for hyperthermia treatment of cancers and tumor masses [19,20]. Polymer-based composites are mainly used in the form of reinforcing elements [21]. One of the best polymers in terms of stability is polyvinyltrimethoxysilane (PVTMS), which it can be synthesized by polymerization of VTMS [22]. The features of the vinyl group (–CH–CH_2_) in the structure of PVTMS can be used to carry out radical polymerization. In addition, PVTMS can improve the thermal stability [23]. Due to the existing voids in HA structures, there are no extreme studies on doping components into HA for sensor applications. Wilson et al. synthesized HA powder and used it for electrochemical sensing of uric acid, and the result was positive when the process had better efficiency [24]. Furthermore, HA is used as a gas detector (alcohol, CO, CO_2_) [25,26,27], as an electrolyte for solid oxide fuel cells [28], as a conditioning matrix for radioactive waste stabilization [29] and for purification of water or soil contaminated with heavy metals [30,31]. Ikoma et al. used HA nanocrystal sensors for protein adsorption and investigated the reusability of the HA sensors and the reproducibility of their measurements for the adsorption of fibrinogen [32]. Laghrouche et al. investigated a highly sensitive humidity sensor based on natural HA [27]. Korostynska et al. manufactured a pressure sensor based on a HA thick film for medical applications [33]. In addition, Fe is the most abundant transition metal in humans and its homeostasis is carefully maintained [34]. Disruption of Fe concentrations can lead to potentially life-threatening conditions [35], so determining the biological availability of Fe is extremely important. In addition, there are several studies on Fe derivatives that have been used based on sensor applications. Goncalves et al. investigated photostable, nontoxic Fe(II) as a sensing device [35]. In other studies, Bucinskas et al. investigated synthetic force sensors based on Fe-(III)oxide and iron oxide powder particles coated with steel and dyes to obtain a tactile sensor system [36]. The surface absorption of oxygen in porous sensing composites was increased due to high porosity. In addition, the strength of these composites decreased when gases such as vaporized ethanol are applied [37]. Due to the continental environment, volatile gases should be reduced, and of the different techniques to determine these toxic gases, gas sensors are desirable. The common phase between the matrix and the reinforcement has a good influence on the performance of the sensor composites. One of the main challenges that can limit the use of sensor composites is the breaking of the bond in the joint between the matrix and the reinforcement [38]; therefore, to prevent this problem, a special freeze-drying machine is fabricated and used in this study. Furthermore, semiconductor metal oxide-based sensors have the disadvantage of high power consumption, short lifetime and instability, so in this study, a new gas/temperature sensor composite consisting of 70% wt. HA, 10% wt. PVTMS and 20% wt. iron(II)chloride tetrahydrate (C_l2_FeH_8_O_4_) is fabricated. The HA serves as the matrix and PVTMS/C_l2_FeH_8_O_4_ as the reinforcement. The chemical structures of HA, PVTMS and C_l2_FeH_8_O_4_ are shown in Figure 1. In addition, X-ray analysis, FTIR, SEM, EDAX, TEM, absorption and photoluminescence measurements, and the response of the sensor to gas and temperature variations are investigated.

## 2. Experimental Methods

### 2.1. Materials and Instruments

In this study, calcium nitrate tetrahydrate (Ca(NO_3_)_2_.4H_2_O), phosphorus pentoxide (P_2_O_5_), iron(II)chloride tetrahydrate (Cl_2_FeH_8_O_4_), and vinyltrimethoxysilane (VTMS) (Merck) were used as the precursors. A mechanical vacuum pump (Hi-Cube) was used in the design of the freeze-dryer. In this study, a new freeze-dryer model was fabricated, which consists of a brass container, thermoelectric cooling (TEC) Eleman, heat sink, fan, Plexi glass, tube (1/4), reservoir, diode bridge, and power supply. The phase series of the powders were confirmed by X-ray diffraction (XRD) and performed on a Philips XRD diffractometer using ${\mathrm{Cu}}_{k\alpha }$ radiation at 40 KV, 30 mA, a step size of 0.05° (2ϴ) and scan rate of 1°/min. In addition, X’Pert software was used for qualitative analysis and reporting of the width of diffraction peaks (rad, β) at full width half maximum (FWHM) in different 2θ values according to the location of the peaks (version 4.9.0). Fourier transform infrared (FTIR) spectroscopy of the composite was performed in potassium bromide (KBr) powder and the instrument was attached with a Perkin-Elmer Spectrum BX FT-IR spectrometer. For the chemical element analyses of the components, an energy dispersive X-ray spectrometer (EDX) Phillips/FEI 149 Quanta 200 was used. In addition, the morphology of the composite was studied using a scanning electron microscope (SEM) Phillips/FEI Quanta 200. Furthermore, transmission electron microscopy (TEM) Tecnai G2 F20 X-TWIN with accelerating voltage from 50 to 80 kV was used. A glove box (vacuum 1 × 10^−6^ Torr) was used and the composite was coated for device fabrication by physical vapor deposition (DS1-170, PVD). A resistance meter ME540 and a heating element made of stainless-steel resistant to acids and corrosives were used to measure the resistance and create the heat generation. Moreover, UV spectroscopy was performed using an AvaSpec-ULS2048XL-EVO and AvaSoft 8. Photoluminescence spectra were recorded using a Hitachi F-4600 FL luminescence spectrophotometer in the wavelength range 400–800 nm; in addition, the wavelength range of the xenon discharge lamp light source used was 250 to 1300 nm.

### 2.2. Fabrication of Freeze Dryer

**Figure 2:**
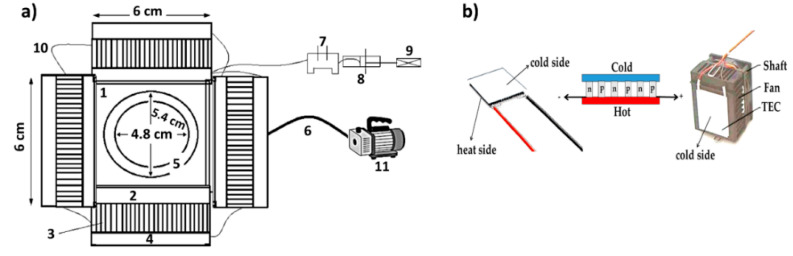
(**a**) Schematic of the freeze-drying system and (**b**) the TEC: interior view of TEC and the connection of the heat side of TEC to the fan via a shaft.

**Figure 3:**
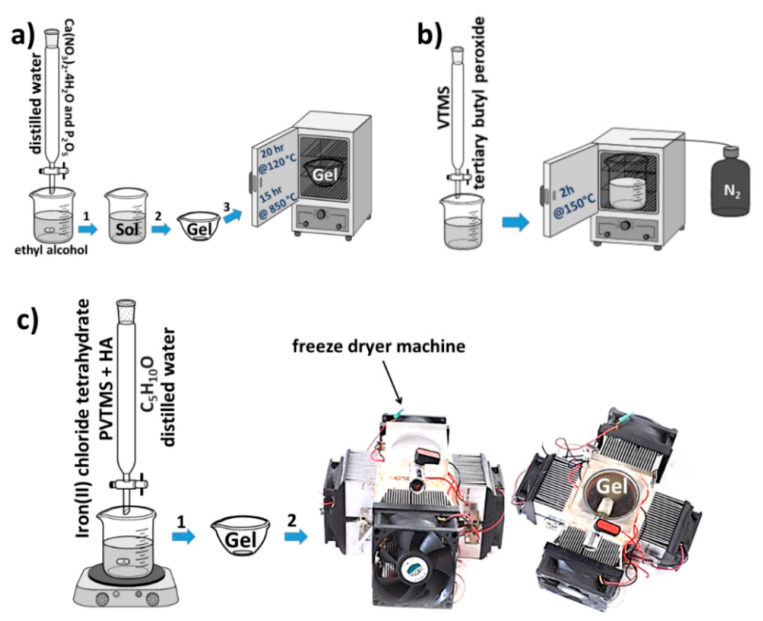
(**a**) Synthesis route of HA, (**b**) polymerization route of VTMS and (**c**) fabrication route of the HA/PVTMS/Cl_2_FeH_8_O_4_ Composite.

Most industrial freeze-drying equipment is used for the production of food and agricultural products, so the use of chemical components, especially those consisting of heavy solvents, in industrial freeze-drying equipment is not common due to the toxicity and the vapor temperature of special components at very low temperatures. In addition, the vapor of chemical volatiles such as carbon, hydrogen and cyclopentyl rings may be damaged during heat treatment, and material collapse may occur during heat treatment. The aim of the design of this freeze-dryer is to vaporize cyclopentyl, water and volatiles in the produced gels and to prevent the collapse and breaking of the bonds, because drying at high temperatures damages the components, especially the polymer. When the target is to prepare a composite as a sensor, it is important to use other methods other than heating to remove the solvents and volatiles. The schematic of the proposed freeze-dryer is shown in Figure 2a. According to the figure, the equipment consists of: (1) a brass container, (2) TEC Eleman, (3) a heat sink, (4) a fan cooler, (5) Plexi glass, (6) tube (1/4), (7) a reservoir, (8) a diode bridge, (9) current, (10) wires and (11) a vacuum pump. Clear images of the freeze-dryer fabricated in this study are shown in Figure 3. Moreover, brass was chosen as the material for temperature transfer, which has a diameter of 5.4 cm. In addition, each TEC is a semiconductor device that can generate a temperature of about −8 °C, and to prevent it from being destroyed, the use of liquid nitrogen is essential. This is because the mechanism of performance has two functions: one side is attributed to cooling and the opposite side is related to heating; therefore, the heating side should be cooled to avoid damage to the TEC. Figure 2b shows the schematic of the TEC and its installation in the system. The heat sink is made of aluminum and its job is related to temperature transfer. Moreover, a fan cooler is responsible for preventing high external temperatures of the TEC. Plexi glass is very transparent and the material is polycarbonate, and its role is to cover the brass fragment and it was also used for the connection to the vacuum pump (insulation). A tube was used to connect the Plexi glass valve to the vacuum pump. The reservoir, diode bridge, current and wires were also connected to the electrical phase. The vacuum pump was used to extract moisture, air and gasses in the non-distilling system. The operating voltage of the device was 15 volts, and the maximum and minimum operating temperatures were 40 °C and −20 °C, respectively.

### 2.3. Preparation of the HA/PVTMS/Cl_2_FeH_8_O_4_ Composite

The purpose of the sol–gel method is to perform chemical processes at low temperatures to produce composites with suitable shapes and surfaces. The sol–gel process can be used to obtain a product size in the range of 1 to 100 nm, which have a structure in the molecular order [39]. In this case, the schematic pathway to synthesis of artificial HA is shown in Figure 3a. Calcium nitrate tetrahydrate (Ca(NO_3_)_2_.4H_2_O) and phosphorus pentoxide (P_2_O_5_) were used as the precursors in a molar ratio of 10:3. The synthesis was as follows: (1) Ca(NO_3_)_2_.4H_2_O and P_2_O_5_ were dissolved in 10 mL ethyl alcohol (C_2_H_5_OH) and distilled water. (2) The product was stirred at 350 rpm for 2 h. (3) The gel was prepared at the bottom of the dish. (4) The gel was then air dried at 120 °C for 20 h. (5) Heat treatment at 850 °C for 15 h was performed for sintering. The sol–gel approaches studied so far have some shortcomings, in particular the use of either expensive alkoxide-based precursors or the need for several complicated steps to ensure complete dissolution of the precursors to produce a pure HA phase after heat treatment. In order to control this problem, more suitable and cheaper precursors were considered in this study as is described in refs. [40,41]. Taking into account the sensor generation, adsorption properties and ion substitutions due to the position of calcium, as well as the fact that both phosphate and hydroxyl can be exchanged by carbonate ions and hydroxyl can also be exchanged by metal ions [27,42,43], HA was considered as the basis of the composite composition. All these features can be associated with the mobility of the charges in the apatite network, which often produces interesting physico-chemical properties. As shown in Figure 3b, vinyltrimethoxysilane (VTMS) was polymerized with a chain length equal to twenty monomers. PVTMS was prepared using tertiary butyl peroxide as an initiator and was refluxed for 2 h at 150 °C under a nitrogen atmosphere [44,45]. Taking into account the depolymerization by an oxygen atmosphere, polymerization was carried out in a nitrogen environment [46]. PVTMS generates a hydroxyl radical (–OH) and the hydrogen radicals (–OOH) are then generated by the reaction of polymerization; these radical species have also been used by SiO_2_/Si. Furthermore, the free radical-mediated oxidation leads to hydroxylation, conversion to carbonyl derivatives and cleavage of the chains, so that deformation and collapse of the composite can be prevented due to the strength of the C chains and free radicals [47,48,49]. In addition, PVTMS contains the functional group silanol (Si-O-H) that can help with bonding; therefore, it is useful for preventing the decomposition of composites [23]. Taking into account the practical applications of α-Fe_2_O_3_ [50], the chloride moieties’ potential as the adsorption–desorption isotherms in sensors, its good sensing, the obvious reaction under evaporated ethanol and the large surface area [50,51], Cl_2_FeH_8_O_4_ was considered as one of the constituents for reinforcement of composite. The products consisting of HA and PVTMS were mixed, and iron(II)chloride tetrahydrate (Cl_2_FeH_8_O_4_) was added simultaneously with cyclopentanol and distilled water and stirred at 140 °C and 450 rpm until gelation was achieved. The sol was converted to a gel, and the gel piece was dried in the fabricated freeze-dryer. After the volatiles, water and solvent were removed, the HA/PVTMS/Cl_2_FeH_8_O_4_ powder composite was prepared.

## 3. Results and Discussion

### 3.1. Study of X-ray Diffraction

**Figure 4:**
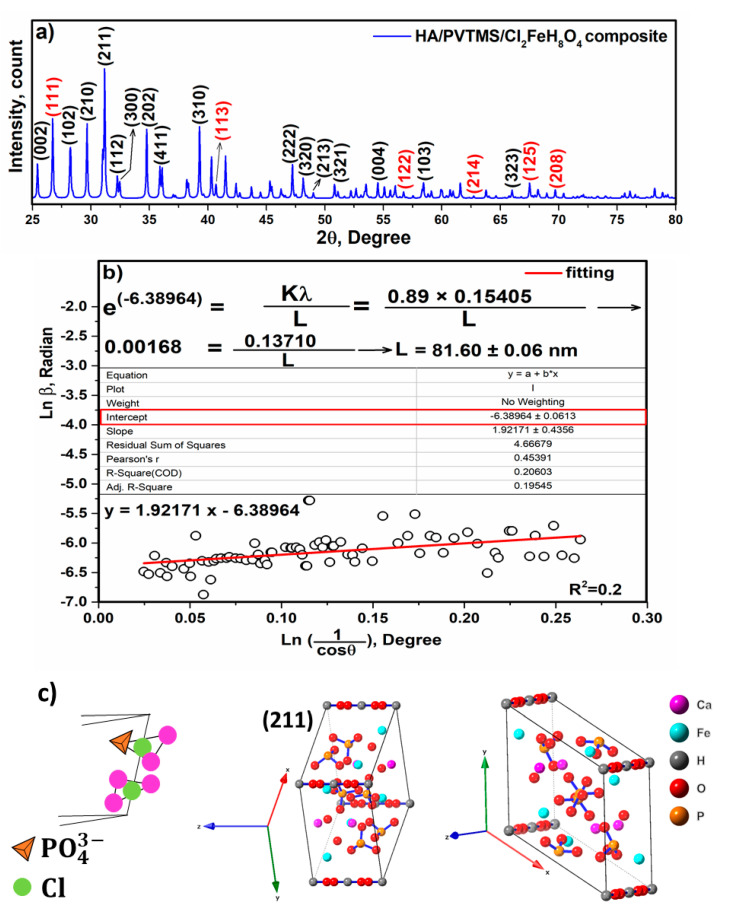
(**a**) X-ray diffraction, (**b**) Linear plots of the Monshi–Scherrer equation, and (**c**) cif file and the view of (211) of the composite.

Figure 4a shows the X-ray diffraction data of the composite, up to 2θ values from 25° to 80°. Phase identification was performed using X-Pert software, and the pattern was consistent with the standard XRD pattern of HA. The black and red peaks represent HA and Fe, respectively. Furthermore, the characteristic peaks at approximately 2θ~31° and 32° are attributed to the (211) and (112) reflections of HA in tandem. The lattice parameter of the composite was recorded to be 9.8931 Å, the value of which was higher than that of non-doped pure HA (~9.4000 Å), this is due to the addition of PVTMS and Cl_2_FeH_8_O_4_, since the radius of Cl is larger than that of O and H. All XRD peaks can be attributed to HA and insignificant amounts of Fe, indicating the absence of other impure phases, while the small peak widths reflect a small size of the crystalline domains. The insignificant Fe peaks are related to the content of the components (20 wt.%), since the Ca/P ratio of 1.6 in the HA phase was taken into account. In addition, the amorphous phase was not observed and the crystal size was calculated using the Monshi–Scherrer method. Scherrer’s base formula (Equation (1)) is presented here, where β is the full width at half maximum of the peak in radians, K is the shape factor, usually assumed to be 0.89 for ceramic materials, λ is the wavelength of the radiation in nanometers ($\lambda_{{\mathrm{Cu}}_{K\alpha }}\;$= 0.15405 nm), ϴ is the diffraction angle of the peak and L is the nanocrystal size [16]. After setting the curve of Figure 4b to Ln ($\frac{1}{\mathrm{Cos}\;ϴ})\;$(Degree) versus Ln β (Radian), the intercept value was determined as −6.38964 and as e^(−6.38964)^ = 0.00168, finally $\frac{K\;\lambda }{L}$ = 0.00168, and the crystal size was gained as L = 81.60 ± 0.06 nm.

(1) $$\mathrm{Ln}\;\beta =\mathrm{Ln}\;(\frac{K\lambda }{L})+\mathrm{Ln}\;(\frac{1}{\mathrm{Cos}\;ϴ})\;$$

The cif file and the (211) plane as a sharp peak of the composite are shown in Figure 4c. The structure of the composite corresponds to a hexagonal system with space group P_63/m_, which is symmetric and perpendicular to the structure of HA due to the higher content of HA than PVTMS and Cl_2_FeH_8_O_4_. The three equivalent axes a, a_2_ and a_3_ form an angle of 120° with each other. In situ, four Ca atoms are surrounded by nine O atoms of phosphate groups belonging to the tetrahedron ${\mathrm{PO}}_{4}^{3-}$. It is noteworthy that the OH ions and four Ca^2+^ ions are located at the Ca sites along columns parallel to the c-axis (Figure 4c), and that some OH ions can be substituted by Cl^-^ and Ca^2+^ ions (second type) and by Fe^2+^ ions located along the c-axis, of which direction of the O–H bond is parallel. It is a valuable point that ions with a higher electronegativity than calcium would be replaced with Ca^2+^ ions (second type) [52]; therefore, Fe^2+^ was substituted with Ca^2+^ ions (second type) due to their high electronegativity (1.83) than Ca (1). These substitutions are described in refs. [53,54,55,56]. In addition, both the phosphate and hydroxyl ions can be replaced by carbonate ions [27].

### 3.2. Investigation of FTIR

**Figure 5:**
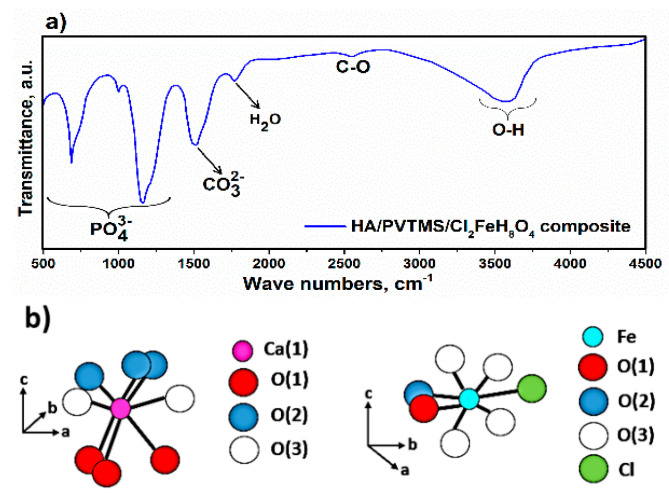
(**a**) FTIR spectrum of composite and (**b**) schematic of Fe and Cl substitution in the composite.

According to the formula of HA, the peaks related to ${\mathrm{PO}}_{4}^{3-}$, ${\mathrm{CO}}_{3}^{2-}$ and OH^−^ are characterized in Figure 5a. The intense bands of ${\mathrm{PO}}_{4}^{3-}$ groups are located at ~633, 856 and 1113 cm^−1^. The small band at 1728 cm^−1^ can be associated with water single stretching; moreover, the relatively broad band from 2700 to 4000 cm^−1^ can be attributed to adsorbed water. The intense IR peaks at 1462 and 2509 cm^−1^ correspond to the C–O band [57,58]. In addition to the HA constituents, the presence of –OH groups have implied the existence of PVTMS, and the small peaks at a wavenumber value of 956 cm^−1^ are caused by iron oxide and PVTMS [59,60]. As a result, according to the FTIR of pure non-doped HA, with addition of the PVTMS and Cl_2_FeH_8_O_4_, the percentages of ${\mathrm{PO}}_{4}^{3-}$ and C–O increase, and the content of hydroxyl groups decreases [61,62,63]. According to FTIR spectra and Figure 5b, the addition of Cl_2_FeH_8_O_4_ into the structure of HA leads to the removal of hydroxyl groups and the development of strain along the c-axis. This strain leads to a higher solubility of HA containing Cl_2_FeH_8_O_4_, as was found in ref. [64].

### 3.3. Study of SEM and TEM Analysis

**Figure 6:**
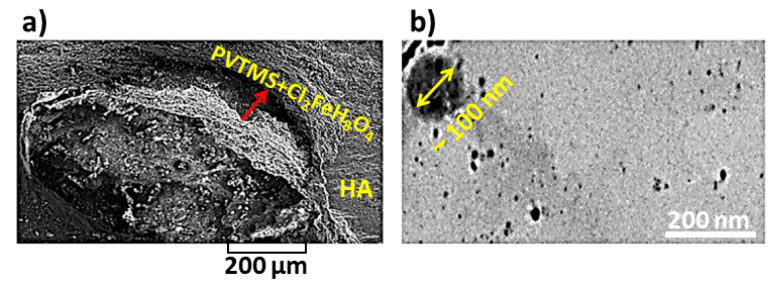
(**a**) SEM and (**b**) TEM images of composite.

The scanning electron microscope (SEM) micrograph of the composite is shown in Figure 6a. The nucleation and growth of PVTMS and Cl_2_FeH_8_O_4_ can be clearly seen on the surface of HA. The microstructure exhibits an irregular morphology but the shells of nucleation initiation at the edges are clearly visible. Moreover, no micro cracks, clusters or agglomerates were observed, while the aggregation of PVTMS and Cl_2_FeH_8_O_4_ particles can be observed on the HA. The chemical constituents in weight percent are listed in Table 1. Table 1 shows that the main elements of the composite are Fe, Cl, P, Ca, O, Si and C with percentages of 12.31, 7.29, 22.21, 33.63, 14.14, 2.38 and 7.00 wt.%, respectively. The presence of copper (Cu) is attributed to the source of the instrument and as a result of extracted elements and contents of EDAX. EDAX was performed to confirm the X-ray diffraction results of the composite when the impurities were not registered. It should be mentioned that the ratio of $\frac{\mathrm{Ca}}{P}$ = $\frac{33.63}{22.21}$, calculated to be 1.51, is not far from the base value of natural HA (1.67) [41,65,66]. The EDAX analysis of the studied composite confirmed the presence of HA as a matrix. The transmission electron microscopy (TEM) image of the composite is presented in Figure 6b. The morphology identifications indicated that nanoparticles were present with a good crystal structure. According to the TEM image, it seems that the crystals are not agglomerated in nanosize particles; the morphology seems to be irregular spheres. Van der Waals attraction [67] was not observed. The grain size determination was carried out from the TEM image, (D_TEM_) is ~100 nm, and this value corresponds to the calculated nano-crystal size extracted by the Monshi–Scherrer method (L = 81.60 ± 0.06 nm). The small difference between (D_TEM_) and L is related to the fact that the images of TEM show the particle size and there are crystals between all particles [16]. This is because the size in TEM is often the same as the particle size [68] and in this study, it is evident that some of the powder particles are nano-sized and the size values are less than 100 nm (diameter).

### 3.4. Investigation of the Behavior of Composite in an Oxygen Environment

**Figure 7:**
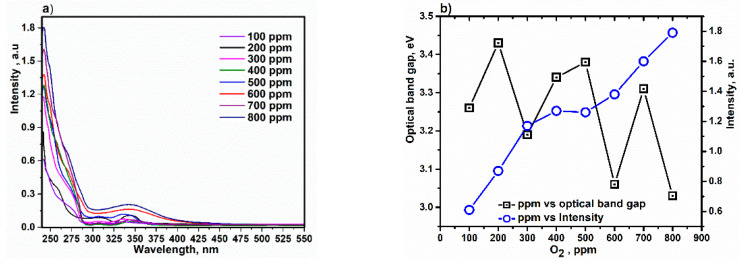
(**a**) Absorption spectra and (**b**) content of oxygen versus absorption intensity and optical band gap of the vapor-coated composite on film.

The UV–Vis spectra and absorption (ABS) behavior of the vapor-coated composite on quartz film are illustrated in Figure 7. The ABS spectra in the range of 240 to 300 nm is almost similar to the natural HA ABS spectra and the maximum ABS wavelength of the composite is ~242 nm [69,70]. In addition, the ABS spectrum in the range from 300 to 425 nm is associated with Cl_2_FeH_8_O_4_ (Figure 7a) [71]. With increasing oxygen concentration, especially at 800 ppm, a slight red shift in the ABS spectra occurs, which is due to the binding of iron(II)chloride to HA [72]. When oxygen is introduced it can be placed in void spaces between hexagons, in the tetrahedral arrangement per unit cell and replaced by OH^−^ ions forming internal channels along the c-axis [73]. As shown in Figure 7a, the intensity of ABS increased with increasing oxygen content, which can be attributed to the charge transfer phenomena between Fe^2+^/Cl^−^ ions and HA based on π→π* transitions similar to ligand to metal charge transfer. This is attributed to the remarkable scattering and absorption of radiation as a result of an increase in surface roughness [74,75]. Moreover, the high absorption of the composite is essentially due to the magnetization of HA due to the existence of Fe ions. As is known, the composite has a higher absorption in the ultraviolet range, and over time the absorption at higher wavelengths (visible region) decreases, which is due to the oxygen gaps in the material [76,77]. In addition, as the oxygen concentration increases (Figure 7b), the electrostatic attraction between the absorbing surface of the composite and the negatively charged ions, such as oxygen and Cl, increases and the intensity increases significantly [78,79]. This occurrence shows that the composite has high efficiency in removing oxygen in the atmosphere due to the existence of magnetic nanoparticles; therefore, the initial properties of the prepared composite show it to be a sensor. The optical band gap (E_g_) values were calculated for each ABS spectrum collected at different oxygen concentrations using the photon energy equation [80], and the range of values were from 3.02 to 3.42 eV. In this case, the increase in E_g_ is due to the minimal filling of the oxygen vacancies in the structure of the composite and the difference between these values can be attributed to quantum size effects associated with the nanocrystallites of the composite [81,82]. Furthermore, the reduction in E_g_ at an oxygen concentration of 800 ppm is due to the fact that the oxygen holes can accept one or two electrons, so the occupied oxygen holes act as donors [83,84].

**Figure 8:**
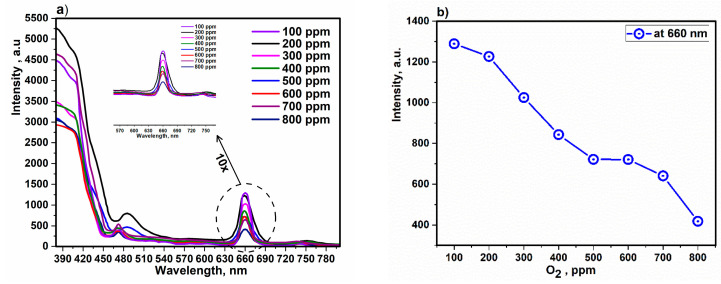
(**a**) PL spectra and (**b**) content of oxygen versus PL intensity of the composite in film mode.

The PL spectrum of the vapor-coated composite on a quartz film and the behavior of the composite with the change in oxygen concentration versus PL intensity at a wavelength of 660 nm are shown in Figure 8a,b. The wavelength range from 450 to 520 nm can be assigned almost entirely to the PL of HA and PVTMS [85], whereas the PL range from 630 to 700 nm corresponds to the Fe moiety [86]. It is noteworthy that as the oxygen concentration was increased, the PL intensity decreased specifically at 660 nm. This is attributed to the structural model of Fe derivatives proposed by Pauling and Hendricks, which suggests a change in the oxygen atomic coordinates in nanoscale α-Fe_2_O_3_ and an increase in the Fe–O bond distance, leading to an enhancement in the coupling of the neighboring atoms, which is also responsible for the photoluminescence [87,88,89]. In another insight, in the composite, Fe derivatives show stronger emission at 660 nm, which is due to the confinement by ionic atoms such as Cl, C and P, which prevent the energy exchange of nanoparticles with the environment and stop the broadening of the electron wavefunction, as well as the surface defects that can be caused by the deep trap created by the vacancy. Therefore, the intensity of PL decreased with increasing oxygen concentration [86,90]. Additionally, a slight shift in the PL spectra is ascribed to the difference in particle and e size and the change in homogeneity and crystallinity with increasing oxygen concentration [91,92].

### 3.5. Fabrication and Investigation Gas/Temperature Sensor Based on Composite

**Figure 9:**
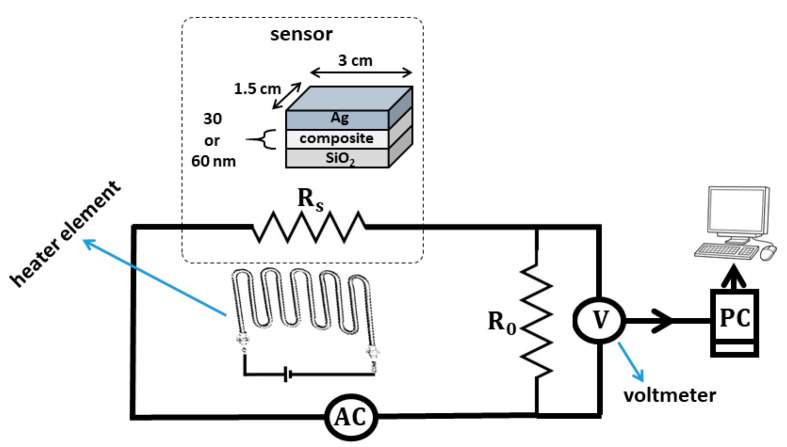
The schematic system for measuring the sensor resistance.

Taking into account that the HA is not completely soluble in solvents, the fabrication of the sensor device was not possible by spin-coating or doctor blade coating methods; hence, the physical vapor deposition (PVD) method was chosen. The area size of the sensor was 3 × 1.5 cm, the active layer thickness was 30 and/or 60 nm and, after stabilizing the connection by silver glue, the device was heated at 80 °C for 10 min. SiO_2_ is one of the best materials due to its suitable features for optical device development, such as high electrical conductivity, wide bandgap energy, transparency in the visible range and suitable thermal and chemical durability; therefore, it was chosen for device fabrication. Furthermore, silver glue was used for metal connections in various quantities. Taking into account the resistance changes measurements changes, a sensor device was developed based on a heating element to raise the temperature of the sensor to the desired working temperature. Then, the Arrhenius curve was used to measure the resistance of the sample as a function of temperature and to determine the resistance of the sensors. The voltage division method was applied so that, according to Figure 9, a fixed resistor in a series circuit was carried out. Knowing the decreasing voltage at constant resistance, the resistance of the sensor can be determined using Equation (2),

(2) $$R_{s}=\left(\frac{V_{\mathrm{in}}}{V_{0}}-1\right)R_{0}$$

where in this equation, $R_{s}$ is the resistance of the sensor at each temperature, $V_{\mathrm{in}}$ is the internal voltage, $V_{0}$ is the voltage across the resistance and $R_{0}$ is the constant resistance in a series circuit with a sensor. With the reading, the constant resistance reduces, and the resistance of the sensor can be determined by Equation (2). In this investigation, ethanol vapor with 1000–5000 ppm concentration was used as the tested gas and changes of resistance with temperature, determination of working temperature and changes in sensitivity according to gas concentration have been evaluated.

According to Equation (3), the exponential pre-factor, which is a reflection of the concentration of charge carriers, is almost constant, in other words, the activation energy, which is a reflection of the kinetic energy, increases with the thickness of the sensor. In this equation, σ is conductivity, $\sigma_{0}$ is constant conductivity, T is temperature, $E_{a}$ is activation energy and K is Boltzmann constant [93,94].

(3) $$\sigma =\frac{\sigma_{0}}{T\exp\left(\frac{-E_{a}}{\mathrm{KT}}\right)}.$$

**Figure 10:**
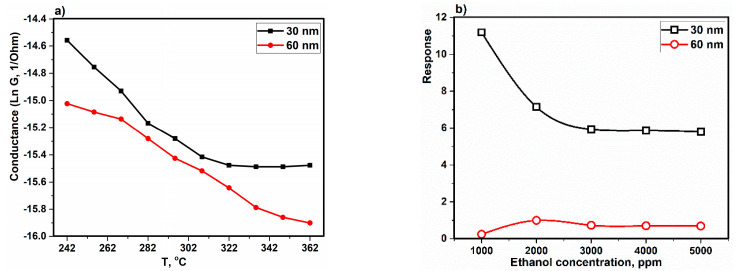
(**a**) Arrhenius curves of sensors with composite layers of thickness of 30 and 60 nm and (**b**) sensitivity of the devices based on ethanol concentrations.

Figure 10 shows the logarithmic of electrical conductivity versus temperature and the dependency of the sensor at the temperature was investigated. Taking into account Equation (3), the activation energy with conductivity has an inverse relationship, and when the activation energy is decreased (slope of curves) the conductivity will be increased [93]. The conductivity of the sensor with 30 nm size is higher than that of 60 nm size due to the lower activation energy. It is worth noting that oxygen molecules are attracted to the surface of the sensor, causing capture of electrons from the conduction band. This creates an oxygen-free surface, causing the barrier to increase the potential and increase the resistance of the sensor [95,96]. In Figure 10b, the response is introduced as response = $\frac{R_{g}-R_{a}}{R_{a}}$, where R_a_ is the sensor resistance in the presence of atmospheric air, R_g_ is the sensor resistance under the gas (evaporated solvent) ambient conditions, and the response is sensitivity in a percentage [97].

**Figure 11:**
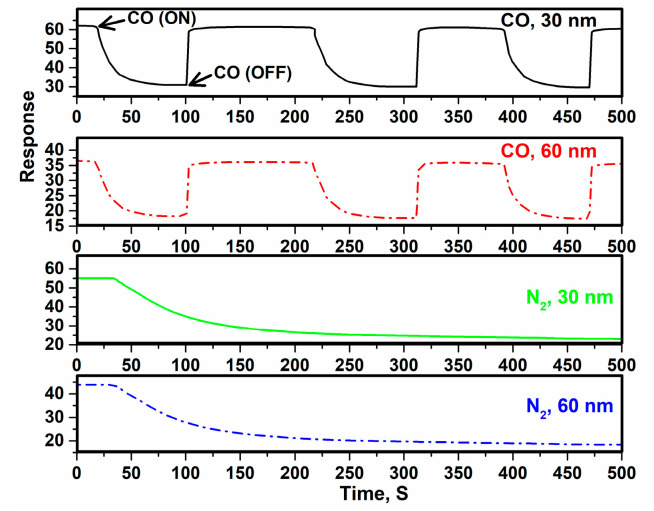
The response of the sensor in the CO and N_2_ environments.

According to Figure 10a, with increasing temperature, the conductivity is increased. That it is related to the features of semiconductor, because the surface oxygen will be picked up via O^2−^, O^−^ and $O_{2}^{-}$ and this process is continued with transfer of electrons and it causes an increase in concentration and decrease in the resistance. In fact, with increasing the temperature, the content of holes and electrons is increased and that causes a decrease in the resistance of the layer [98]. Furthermore, with increasing the thickness of the composite layer, the activation energy is increased because the slope of curve at 60 nm is increased; therefore, the diffusion is decreased. In addition, the band gap is decreased when the thickness of the composite layer is decreased to 30 nm; therefore, the Fermi level is getting closer to the highest level of the valence band, so the acceptor atoms have changed to more superficial states and their energy of ionization is reduced and needs the less activation energy. Moreover, with increasing gas concentration, the reaction between gas molecules and oxygen atoms increases due to surface absorption. Therefore, with increasing the concentration of ethanol gas, the sensitivity will be increased. When the maximum sensitivity occurs (saturated phase), all oxygen ions will be used in the reaction, and with increasing the concentration above the optimum value (saturated phase), the sensitivity is decreased. According to Figure 11, the device with a 30 nm-thick composite has the maximum response of the sensor due to more absorption of gas and the interaction between gas molecules and the surface being stronger [99]. The slow recovery is due to the strong attraction between the surface and the target molecules via hydrogen bonds, which requires several minutes to return the substrate to its initial state under atmospheric conditions. In addition, there is a higher presence of oxygen holes in the device with a 30 nm-thick composite, which leads to a decreased band gap and an increase in electron transfer, so the resistance is higher than in devices with a thickness of 60 nm, as was found in ref [84]. Furthermore, it is clear that the sensitivity of the sensors decreases with time, especially in the N_2_ environment. The reason is related to the decrease in oxygen on the surface, as the composite is very sensitive to the absorption of oxygen (Figure 7 and Figure 8), so there is not enough oxygen to react with the gas molecules [74,100]. As the thickness of the active layer increases, the direct interaction between the gas molecule and the composite surface is limited so that the physical adsorption of gas molecules on the surface is limited.

## 4. Conclusions

A sensor composite consisting of HA/PVTMS/Cl_2_FeH_8_O_4_ was designed because the initial HA and iron components were sensitive to temperature and gas, and PVTMS led to strong binding due to the presence of Si moieties in the structure. Therefore, HA was used as matrix and PVTMS and Cl_2_FeH_8_O_4_ were used as reinforcements in the composite. The composite was successfully synthesized by the sol–gel method. The leakage of volatiles and water from the composite was challenging due to the collapse and breaking of bonds during heat treatment due to the sensitivity of HA to high temperature; therefore, a piece of special freeze-drying equipment was designed and fabricated to produce the composite. The crystal structure of the composite was studied by X-ray diffraction. The system was hexagonal and the addition of PVTMS and Cl_2_FeH_8_O_4_ did not change the crystal system when the weight percentage of HA was 70 wt.%. The crystal size of the composite was calculated by the Monshi–Scherrer method to be 81.60 ± 0.06 nm, which was consistent with the values extracted from TEM analysis (less than 100 nm). Moreover, the FTIR analysis mainly showed the ${\mathrm{PO}}_{4}^{3-}$ and C–O components of HA and proved that there were no impurities in the composite structure. The SEM of the composite was investigated and micro-cracks, clusters and agglomerates were not observed when the $\frac{\mathrm{Ca}}{P}$ ratio was calculated to be 1.51, proving the existence of HA. The maximum ABS wavelength of the vapor-coated composite on quartz film in an oxygen environment was measured at ~242 nm. Additionally, the optical band gap (E_g_) values were determined to be in the range of 3.02 to 3.42 eV. Furthermore, the PL spectrum of the vapor composite deposited on quartz film in an oxygen atmosphere showed peaks in the wavelength range from 450 to 520 nm and the range from 630 to 700 nm, which corresponded to PL of HA/PVTMS and the Fe moiety, respectively. The sensor device SiO_2_/composite/Ag was fabricated by the PVD method and with silver glue. In addition, the gas/temperature sensing performances of devices with a 30 nm- and 60 nm-thick layer of composite were evaluated and compared, and the conductivity and sensitivity increased with increasing temperature. Based on ethanol concentrations, the sensitivity of the sensor with a 60 nm-thick layer of composite was lower than that of the 30 nm-thick composite device. In addition, the resistance of the sensor was investigated in CO and N_2_ environments, and the resistance response of the device with a 30 nm-thick composite layer was higher than that of the 60 nm-thick layer composite device. Overall, the HA/PVTMS/Cl_2_FeH_8_O_4_ composite was characterized and investigated, and the results showed that this type of composite, as well as the sensor device fabricated based on the composite, were sensitive to changes in gas and temperature parameters.

## Figures and Tables

**Table 1:** The weight % of composite ingredients based on EDAX analysis.

Number	Element	Weight %
1	Fe	12.31 ± 0.01
2	Cl	7.29 ± 0.01
3	P	22.21 ± 0.01
4	Ca	33.63 ± 0.01
5	O	14.14 ± 0.01
6	Si	2.38 ± 0.01
7	C	7.00 ± 0.01
8	Cu	1.04 ± 0.01

## Data Availability

Data sharing is not applicable.
